# Targeted Release of Probiotics from Enteric Microparticulated Formulations

**DOI:** 10.3390/polym11101668

**Published:** 2019-10-13

**Authors:** Cristina Yus, Ruben Gracia, Ane Larrea, Vanesa Andreu, Silvia Irusta, Victor Sebastian, Gracia Mendoza, Manuel Arruebo

**Affiliations:** 1 Department of Chemical Engineering, Aragón Institute of Nanoscience (INA), University of Zaragoza, Campus Río Ebro-Edificio I+D, C/ Poeta Mariano Esquillor S/N, 50018-Zaragoza, Spain; cyargon@gmail.com (C.Y.); r.gracia1992@gmail.com (R.G.); ane.larreaibanez@gmail.com (A.L.); vandreu@unizar.es (V.A.); sirusta@unizar.es (S.I.); victorse@unizar.es (V.S.); arruebom@unizar.es (M.A.); 2 Aragon Health Research Institute (IIS Aragon), 50009 Zaragoza, Spain; 3 Networking Research Center on Bioengineering, Biomaterials and Nanomedicine, CIBER-BBN, 28029-Madrid, Spain

**Keywords:** polymethacrylate, Eudraguard, Eudragit, probiotics, target delivery, enteric coating

## Abstract

The development of advanced probiotic delivery systems, which preserve bacteria from degradation of the gastrointestinal tract and achieve a targeted release mediated by pH-independent swelling, is of great interest to improve the efficient delivery of probiotic bacteria to the target tissue. Gram-positive and Gram-negative bacteria models (*Lactobacillus acidophilus* (Moro) Hansen and Mocquot (ATCC® 4356™) and *Escherichia coli* S17, respectively) have been successfully encapsulated for the first time in pH-independent microparticulate polymethacrylates (i.e., Eudraguard biotic) used for the targeted delivery of nutraceuticals to the colon. These bacteria have also been encapsulated within the mucoadhesive polymethacrylate Eudragit RS 100 widely used as targeted release formulation for active pharmaceutical ingredients. The enteric microparticles remained unaltered under simulated gastric conditions and released the contained viable microbial cargo under simulated intestinal conditions. Buoyancies of 90.2% and 57.3% for Eudragit and Eudraguard microparticles, respectively, and long-term stability (5 months) for the encapsulated microorganisms were found. Cytotoxicity of the microparticles formulated with both polymers was evaluated (0.5–20 mg/mL) on Caco-2 cells, showing high cytocompatibility. These results underline the suitability of the synthesized materials for the successful delivery of probiotic formulations to the target organ, highlighting for the first time the potential use of Eudraguard biotic as an effective enteric coating for the targeted delivery of probiotics.

## 1. Introduction

Copolymers of methyl acrylate, methyl methacrylate, and methacrylic acid are used as enteric coatings on active principles or as blends to protect them from the degradation of the gastrointestinal tract. They are administered as oral solid dosage formulations in the form of binders or as coatings on different substrates containing the active pharmaceutical ingredient. Enteric coatings are industrially produced using pan coaters or fluidized bed coaters. In the fabrication suspensions composed of the polymer, plasticizers and anti-tacking agents are sprayed and dried on the specific substrate forming an enteric protective covering [1].

Those polymers remain protonated and consequently insoluble under the acidic gastric conditions and they dissolve or erode releasing the contained cargo under intestinal conditions [2]. Some of them are pH responsive and dissolve by salt formation when reaching a specific pH. Others, thanks to their chemical modification (e.g., with quaternary ammonium groups), attach to the intestinal mucosa lining the intestinal epithelial tissues and, although insoluble, they slowly permeabilize and erode providing with a time-controlled targeted release of the contained cargo. 

Different therapeutic molecules have been encapsulated within those acrylic polymers to protect them from the enzymatic (mainly pepsin and lipase) and acidic (pH ≈ 1.5–4) degradation that takes place in the stomach. In this regard, different drugs have been encapsulated within nanoparticles based on the polymethacrylate polymer Eudragit S100 (pH responsive formulation intended for colon delivery) [3,4], on Eudragit L100–55 (pH sensitive polymethacrylate) [5,6], or on Eudragit FS30 D (pH > 7: responsive enteric formulation) [7], showing improved pharmacokinetics and targeting ability.

Polymethacrylate-based polymers can be used not only to prevent pharmaceuticals from gastric degradation but also nutraceuticals, including dietary supplements and pre- and probiotics. Eudragit S100 has been used to encapsulate nicotinamide, being the resulting nanoparticles efficient antimicrobial agents [8].

Non-dairy probiotics are commonly commercialized as lyophilized bacterial powders containing the cryoprotectants (i.e., sucrose, lactose) used during the freeze-drying process and anti-tacking agents (i.e., magnesium stearate, silica, etc.) to avoid adherence to the manufacturing equipment and to prevent agglomeration. Some bacteria strains (i.e., *Lactobacillus acidophilus*, *Bifidobacterium*, *Streptococcus mutants*, etc.) partially resist the acidic environment of the stomach and the high bile salt conditions of the intestine, but others (i.e., *Lactobacillus delbrueckii*, *Streptococcus thermophiles*, *Escherichia coli Nissle* 1917, etc.) require their protection using enteric coatings [9,10]. Compared to the use of large capsules, microencapsulation of live bacteria has the advantage of reducing the carrier residence time in the stomach thanks to the reduced micrometric particle size. In addition, due to the large area per volume ratio of microparticles, they might produce an efficient and homogeneous distribution of the encapsulated active principle on the intestinal surface area. Microencapsulation of bacterial cells using alginate, chitosan, cellulose derivatives, shellac, etc., has been developed to protect bacteria from degradation, re-populate the gut microbiota, and to achieve a targeted delivery [11]. Polymethacrylate polymers containing live bacteria have been primarily applied as coatings on millimetric pellets [12,13], or discs [14], or as compressed bacterial tablets using Eudragit as binder [15].

There is a growing body of evidence of the health benefits induced by pre- and probiotics supported by standardized double-blinded randomized clinical trials for the treatment of different pathologies including irritable bowel syndrome [16,17,18]; chronic idiopathic constipation [19,20,21]; diarrhoea in patients treated with radiotherapy [22]; necrotizing enterocolitis [23]; and in protecting the intestinal mucosa barrier in patients with colorectal cancer [24]. However, most of those meta-analyses conclude that further research is needed with the goal of identifying the most effective strains, dosages, treatment duration, and the magnitude of the improvement.

In order to shed light on this field, advanced probiotic delivery systems, which preserve bacteria from degradation, need to be developed. Therefore, herein we report for the first time the successful microencapsulation and improved survival of model bacteria (*E. coli* as Gram negative bacteria [25] and *L. acidophilus* as Gram positive bacteria) within a commercial polymethacrylate, Eudraguard biotic, mainly used for the targeted delivery of nutraceuticals to the colon. To the best of our knowledge, this is the first work that reports the suitability of this polymethacrylate for the successful targeted delivery of probiotics. We also used the mucoadhesive polymethacrylate Eudragit RS 100, widely used as targeted and time controlled release formulation by pH-independent swelling, for active pharmaceutical ingredients. In most of previous reported works, Eudragit is applied as a top layer on pellets, particles, or coatings to provide the loaded bacteria with gastroresistance and with targeting ability to a specific part of the intestine. In this work, as a novelty, the complete microparticle and not only the top layer, is composed of Eudragit acting as a reservoir, having mucoadhesive properties and targeting ability. In addition, to the best of our knowledge, this is the first manuscript reporting the use of Eudraguard for the encapsulation of probiotics and the use of complete Eudragit microparticles for probiotic targeted delivery.

## 2. Materials and Methods

### 2.1. Reagents

Eudragit RS 100 and Eudraguard biotic were gently donated by Evonik Industries AG (Essen, Germany). Chloroform, poly(vinyl alcohol) (PVA, MW: 85,000–124,000 Da), triethyl citrate (TEC), Fluorescein 5-isothiocyanate (FITC), acetic acid, and sodium hydroxide were purchased from Sigma-Aldrich (Darmstadt, Germany) and used as received. Tryptone soy broth (TSB), MRS broth, MRS agar, and tryptone soy agar (TSA) were purchased from Laboratorios Conda-Pronadisa SA, (Torrejon de Ardoz, Spain).

*E. coli* S17, as a Gram negative bacteria model, was kindly donated by Dr. J.A. Ainsa (University of Zaragoza, Zaragoza, Spain), while *L. acidophilus* (Moro) Hansen and Mocquot (ATCC® 4356™) as a Gram positive model was purchased from LGC group (Barcelona, Spain), both contained glycerol as a cryoprotectant. Simulated gastric (HCl (0.1 N), NaCl, and H_2_O, pH 1.1) and intestinal fluids (TSB, pH 7.4) were also used [26,27].

### 2.2. Synthesis of Enteric Microparticles Containing Probiotic Bacteria

The high internal phase emulsion (HIPE) method was followed in order to generate the enteric microparticles with sizes lower than 250 µm, which have been reported to facilitate larger intestinal residence times [28]. In order to obtain these sizes, different solvents (i.e., ethanol, dichloromethane), surfactants (i.e., polyethylene glycol, polyvinyl alcohol) or polymer concentrations, among others, were varied in order to match the desired size (Table S1).

The inner aqueous phase of the HIPE was composed of the corresponding bacteria dispersed in TSB. In this regard, an *E. coli* S17 colony was inoculated in 4 mL of TSB and incubated overnight at 37 °C, under stirring at 150 rpm. Afterwards, the resulting bacteria in their exponential phase of growth (10^6^ CFU/mL) were centrifuged (5 min, 3000 rpm) and the resuspended pellet was dispersed in 4 mL of TSB acidified with acetic acid to reach pH 4.3 in order to avoid the polymer degradation at neutral pH. For the encapsulation of *L. acidophilus*, 1 mL of bacteria (10^6^ CFU/mL) in 10 mL of MRS broth was incubated overnight at 37 °C under stirring at 150 rpm. Afterwards, the resulting bacteria in their exponential phase of growth (10^6^ CFU/mL) were centrifuged (5 min, 3000 rpm) and the resuspended pellet was dispersed in 4 mL of MRS broth acidified with acetic acid to reach pH 4.3.

The organic phase was composed of 100 mg Eudraguard biotic or Eudragit RS 100 and 2% (*w/v*) of TEC dissolved in 1 mL of chloroform.

HIPE, as previously reported [29,30,31], was formed by mixing under stirring (5 min, 800 rpm) the organic phase with the aqueous bacteria-containing inner phase (4 mL) at 4 °C. The HIPE was immediately stabilized in a second emulsion with PVA dissolved in TSB (2 mL, 1% (*w/v*), pH 4.3) under stirring (5 min, 800 rpm). The double emulsion, constituted of high internal phase emulsion (DE-HIPE), was stabilized by adding a PVA solution in TSB (10 mL, 0.3% (*w/v*), pH 4.3). Magnetic stirring at 600 rpm on an ice bath was maintained for 3 h to allow the evaporation of the organic solvent and the subsequent polymer precipitation as micron-sized particles. The presence of TEC was required to avoid the formation of microparticles with a porous structure and to protect bacteria under gastric conditions. The resulting microparticles were thoroughly washed by decantation replacing the supernatant with acidified DDI water (pH 4.3) for three times.

The same protocol was followed to encapsulate FITC as a model to optically analyze the potential release of the encapsulated dye under simulated gastric and intestinal conditions. The inner aqueous phase was in this case replaced by a water solution of FITC 0.025% (*w/v*) at a pH of 4.5. Empty particles were also prepared during the synthesis optimization process as control samples. 

Furthermore, in vitro drug release kinetics were evaluated by dispersing FITC-loaded microparticles in simulated gastric fluid at 37 °C for 2 h. Then, those microparticles were centrifugated and the supernatant was discarded, and the gastric fluid replaced by simulated intestinal fluid at 37 °C. At specific time points (2, 4, 6, 8, 24, 48 h) samples were collected and the FITC released was quantified by UV-VIS spectrophotometry (absorption maximum centered at 480 nm). Data were fitted to the Korsmeyer–Peppas [32] and Higuchi [33] models to obtain the kinetic parameters using the following equation:

*M*_t_/*M*_∞_ = k·*t*^n^ (1)

where k is the constant related to the characteristic of the polymeric matrix and n the diffusional exponent whereas M_t_ is the drug release fraction at time t and *M*_∞_ is the total amount of drug released until the particles are exhausted.

Correlation coefficient (R^2^) values were determined from the linear regression of the plots.

### 2.3. Microparticles Characterization

An Inspect F50 field emission gun scanning electron microscope operated at 5 and 10 kV was used to visualize the morphology of the resulting microparticles. Those were previously Au/Pd coated (Leica EM ACE200, Leica; Wetzlar, Germany) to allow electronic observation. Particle-size distribution was calculated using a statistical analysis of the SEM images (N = 150). Thermogravimetric analysis (Mettler Toledo TGA/STDA 851e, Mettler Toledo; Columbus, OH, US) was performed to quantify the bacterial loading within the polymeric materials based on the different degradation temperatures of the microorganisms and the ones of the enteric polymers. We initially evaluated separately the decomposition temperatures of both bacteria and polymer (Eudragit RS 100 or Eudraguard biotic) to be able to decouple one from the other and quantify the amount of bacteria loaded inside the microparticulated polymeric carriers. Samples were analyzed in air (gas flow 50 mL/min) in a temperature range between 30 and 800 °C with a heating rate of 10 °C/min. A faster weight loss occurring for the bacteria at 225 °C compared to the weight losses accounted for the enteric polymers was used to evaluate the amount of encapsulated bacteria.

In order to quantify the floating behavior, a known quantity of both Eudraguard and Eudragit RS 100 microparticles was placed in simulated gastric fluid (pH 1.1, 5 mL). After 8 h under magnetic stirring at 200 rpm, the particles remaining on the surface were collected and dried at 60 °C until reaching constant weight. Buoyancy was quantified by the ratio between the weight of the floating particles remaining at the surface and the total weight of the initial particles.

Mass balance was determined by the weight ratio of the lyophilized microparticles, in triplicated, over the total mass of all the reagents used in the synthesis.

### 2.4. Microbiological Studies

The influence of the synthesis protocol and reagents used in the bacterial viability was analyzed by contacting the bacteria with the independent reagents (TSB at pH 4.3; TEC at 2% (*w/v*); PVA 1% (*w/v*)). Also the influence of the physical conditions used (ice bath for 3.5 h and magnetic stirring for 3.5 h) on the bacteria viability was evaluated. The serial dilution method in PBS was used to count viable bacteria. All the results were calculated in triplicate using two independent syntheses.

The dispersion of the encapsulated dye or bacteria from the enteric microspheres was studied in simulated gastric fluid (2 h) using the paddle method under sink conditions. The simulated gastric fluid was prepared with water when analyzing the potential diffusion of the dye to the media and it was prepared in TSB (for *E. coli*) and in MRS (for *L. acidophilus*) when studying the potential release of viable bacteria to the media. Those specific media were used in order to allow bacterial growth. After 2 h the supernatant was collected and plated to count viable bacteria. To analyze the viability of the bacteria under intestinal conditions, the bacterial pellet collected after the 2 h used for simulating gastric conditions was placed under sink conditions in TSB basified with NaOH (pH 7.4) for 24 h or 48 h (with medium replacement) to mimic intestinal conditions. Again the supernatant was plated and viable bacteria counted using the serial dilution method. The experiments were run in triplicate using three independent syntheses.

### 2.5. Long-Term Stability Studies

Long-term stability was also assessed by contacting previously lyophilized Eudraguard or Eudragit-encapsulated bacteria with gastric and intestinal fluids as previously described. With this aim, the bacteria collected after one synthesis was lyophilized (LyoQuest, Telstar <0.1 mBar, −50 °C, 24 h) without the addition of any additional cryoprotectant. Once lyophilized the microparticles were stored on a laboratory bench 22 ± 3 °C and at 43% ± 7% of humidity (mean of 10 days). After different times (4 days, 1 month, and 5 months) the microparticles were reconstituted in 10 mL of TSB (pH 7.4 balanced with NaOH) for *E. coli* or in 10 mL of MRS for *L. acidophilus* and incubated during 24 h at 37 °C. In some cases, after 24 h, 10 mL more of fresh media were added and the particles were incubated for 24 h at 37 °C. Recovered colonies were counted using the serial dilution method. Again, the results were obtained from three independent syntheses and bacterial encapsulation tested in triplicate.

### 2.6. In Vitro Cell Viability Assay

TC7 clone human epithelial colorectal adenocarcinoma (Caco-2) cells, kindly donated by Dr. M.J. Rodriguez Yoldi, were grown at 37 °C in a humidified atmosphere containing CO_2_ (5%) and under hypoxic conditions (3% O_2_). Caco-2 cells were cultured in Dulbecco’s modified Eagle’s medium high glucose (DMEM w/stable glutamine; Biowest, France) supplemented with 10% (*v/v*) fetal bovine serum (FBS; Thermo Fisher Scientific) and 1% penicillin/streptomycin/amphotericin B (Biowest).

The cytotoxicity effect of Eudragit RS 100 and Eudraguard biotic microparticles were studied in Caco-2 TC7 cells using Blue Cell viability assay kit (Abnova), according to the recommended manufacturer´s protocol. For that, cells were seeded at a density of 1.5 × 10^4^ cells per well in 96-well plates, and allowed to attach and proliferate until reaching 100% confluence.

For cytotoxicity screening experiments, cells were exposed to a concentration range of enteric microparticles (0.5–20 mg/mL) in complete growth medium for 24 h at 37 °C. After incubation, cells were washed twice with Dulbecco’s phosphate buffered saline (DPBS, Biowest) and incubated with DMEM containing 10% (*v/v*) the Blue Cell viability reagent (Abnova) for 4 h.

Fluorescence was measured at excitation and emission wavelengths of 530 nm and 590 nm, respectively, in a multi-mode Synergy HT Microplate Reader (Biotek). Cell viability was expressed as a relative percentage compared to the one retrieved for untreated cells. The percentages obtained depict the average of eight independent values.

### 2.7. Statistical Analyses

All results are expressed as mean ± standard deviation (SD). Normal distribution of the variables and one-way analysis of variance (ANOVA) were used to analyze bacteria countings (Minitab Software 17.1.0). Statistically significant differences among groups were considered when *p* ≤ 0.05.

## 3. Results and Discussion

### 3.1. Synthesis and Characterization of Fabricated Microparticles

**Figure 1:**
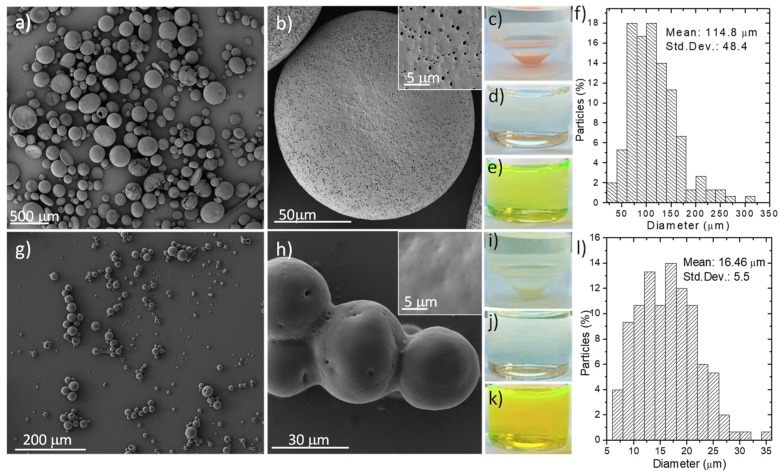
SEM photographs showing the morphology of empty microparticles based on: (**a**,**b**) Eudragit RS 100; (**g**,**h**) Eudraguard biotic; (**c**) Optical imaging of an Eudragit-based microparticle suspension in water; (**d**) Optical imaging of the supernatant collected from a Eudragit-based microparticle suspension after 2 h at 37 °C under simulated gastric conditions; (**e**) Optical imaging of the supernatant collected from a Eudragit-based microparticle suspension after 6 h at 37 °C under simulated intestinal conditions; (**f**) Size distribution of Eudragit RS 100 microparticles obtained (N = 150); (**i**) Optical imaging of a Eudraguard-based microparticle suspension in water; (**j**) Optical imaging of the supernatant collected from a Eudraguard-based microparticle suspension after 2 h at 37 °C under simulated gastric conditions; (**k**) Optical imaging of the supernatant collected from a Eudraguard-based microparticle suspension after 6 h at 37 °C under simulated intestinal conditions; (**l**) Size distribution of Eudraguard biotic microparticles obtained (N = 150). Std. Dev. = Standard Deviation.

Figure 1 shows the morphology of the empty enteric microparticles. Eudraguard biotic rendered particles much smaller (16.5 ± 5.5 µm; Figure 1l) than the ones obtained when using Eudragit RS 100 (114.8 ± 48.5 µm; Figure 1f). This particle-size distribution was calculated using a statistical analysis of the SEM images (N = 150). The different polymer composition, pH, and viscosity of Eudraguard and Eudragit might be responsible for obtaining different particle sizes during the emulsion solvent evaporation process. According to the manufacturer, Eudraguard biotic showed 9.6% of methacrylic acid units based on dried solid (DS), an alkali value of 63 mg KOH/g DS (caused by its carboxylic pending groups), and an apparent viscosity of 4 mPa·s whereas Eudragit RS 100 showed a 5.5% ammonium methacrylate units on DS, an alkali value of 14.7 mg KOH/g DS, and an apparent viscosity of 6 mPa·s. The higher apparent viscosity of Eudragit might be responsible for a larger particle size due to the reduction in the shear stress caused during the mechanical emulsification process in agreement with the previous literature [34]. Eudragit-based microparticles showed pores (Figure 1b) on their surface even with the addition of TEC as a plasticizer. Eudraguard-based microparticles showed a smooth surface with no pores, just a few dimples (Figure 1h). However, dye-diffusion studies using FITC quantitatively showed that both Eudragit- and Eudraguard-based microparticles released only a 3.6 and 1.2 wt %, respectively, of the encapsulated dye after 2 h of immersion in simulated gastric fluid (Figure S1). Therefore, the fluid was not able to penetrate within the polymeric structure under the conditions studied. As predicted, the particles released the contained dye after immersion in simulated intestinal fluid (Figure 1e,k). Furthermore, dye release kinetics indicated that FITC released from Eudragit RS 100 microparticles following a Higuchi model (% released = K × *t*^0.5^), showing as release parameters n = 0.46 (≈0.5) and *R*^2^ = 0.92. Eudraguard-based microparticles exerted FITC release kinetics that could be fitted to an anomalous diffusion model (partial diffusion through a swollen matrix) displaying parameter values of n = 0.07 (≈0.1; n < 0.5) and *R*^2^ = 0.93. The microparticles showed a buoyancy of 90.2% and 57.3% for Eudragit and Eudraguard, respectively, which is indicative of their suitability for achieving large residence times in the gastrointestinal tract.

**Figure 2:**
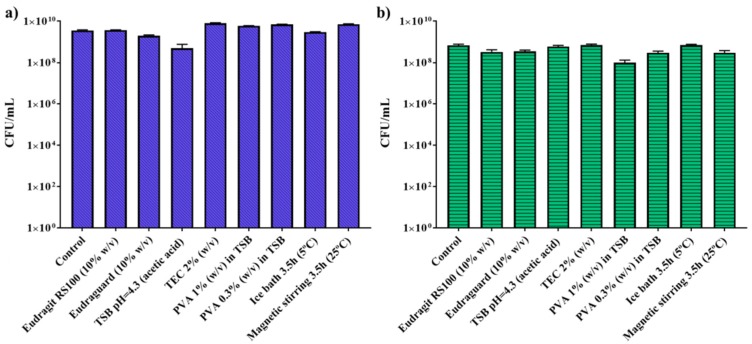
Bacteria viability after polymers, encapsulation media, and synthesis conditions treatment in *Escherichia coli* (**a**) and *Lactobacillus acidophilus* (**b**). Results were obtained from two independent syntheses assayed in triplicate.

Figure 2 shows that the polymer, reactants, and synthesis conditions (low temperature (4 °C) and mechanical stirring) did not impair bacterial replication and cell counts similar to the ones obtained for the control were obtained. Chloroform is part of the organic phase during the synthesis, however, due to its immiscibility with water the contact between chloroform and the aqueous phase containing the bacteria would be minimum corroborating the lack of cytotoxicity observed. In addition, in the case of TEC, it might be displaying a protective effect on bacteria regarding the cryoprotectant role of citrate groups [35].

**Figure 3:**
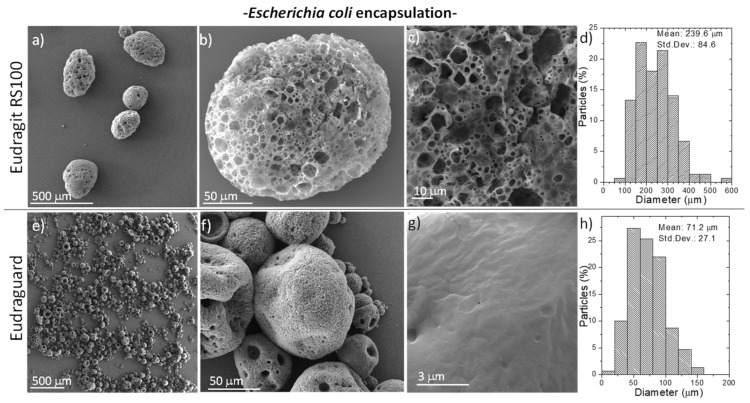
SEM micrographs (**a**–**c**,**e**–**g**) with different magnifications and particle size distribution histograms (**d**,**h**) (N = 150) of Eudragit RS 100 (**a**–**d**) and Eudraguard (**e**–**h**) microparticles loaded with *Escherichia coli*. Std. Dev. = Standard Deviation.

**Figure 4:**
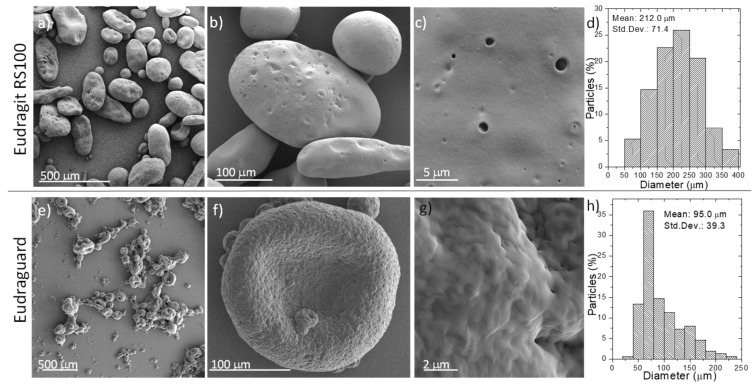
SEM micrographs (**a**–**c**,**e**–**g**) with different magnifications and particle size distribution histograms (**d**,**h**) (N = 150) of Eudragit RS 100 (**a**–**d**) and Eudraguard (**e**–**h**) microparticles loaded with *Lactobacillus acidophilus*. Std. Dev. = Standard Deviation.

When bacteria were encapsulated within the enteric microparticles, the resulting particle size increased compared to the one of the empty ones (Figure 3 and Figure 4). Thus, the size of the Eudragit-based microparticles increased to 239.6 ± 84.6 µm when *E. coli* was encapsulated (2-fold increase compared to the empty ones) (Figure 3). Eudraguard-based microparticles also increased their size when *E. coli* was encapsulated within them (4-fold increase from the initial 16.5 ± 5.5 µm, to the final 71.2 ± 27.1 µm when the particles were loaded with bacteria) (Figure 4). Also, when *L. acidophilus* was encapsulated within the Eudragit-based microparticles, the size increased even more reaching 212.0 ± 71.4 µm (Figure 3) and within Eudraguard-based microparticles a final particle size of 95.0 ± 39.3 µm was measured (Figure 4). Consequently, it seems that the presence of bacteria with lengths higher than 2.5 µm (Figure S2) might be responsible for the increase in the loaded particle size. Besides, bacteria growing media viscosity is higher than water viscosity and this increases with bacterial growth [36]. The higher viscosity of the inner phase could also contribute to the larger size of the loaded microparticles [34].

The surface porosity also seems to be dependent on the different bacteria encapsulated, showing an enhanced surface porosity when *E. coli* was encapsulated within Eudragit-based microparticles compared to the encapsulation of *L. acidophilus*. Again, for the same encapsulated bacteria the surface roughness was reduced when using Eudraguard compared to Eudragit. It is important to point out that all the microparticulate systems here developed showed sizes below 250 micron, which are recommended to reach large residence time in the intestine avoiding a rapid gastrointestinal excretion [28]. Also, microparticles with sizes above a few microns are largely excluded from systemic absorption [37] and therefore the potential uncontrolled bacteremia would be largely hindered. Another advantage of microparticulated carriers compared to the use of hard or soft capsules coated with enteric coatings is that local irritation is largely prevented and pharmacokinetics are easier to reproduce.

Bacteria encapsulation efficiency was calculated by using TGA (Figure S3) analysis revealing bacteria loadings of 2.5 wt % in Eudragit RS 100 and 2.8 wt % in Eudraguard encapsulation. Food industry recommends 10^6^ CFU/mL of *L. acidophilus* per gram of product with the aim of exerting beneficial effects on human health [38], being 10^8^–10^9^ CFUs the recommended minimum effective dose per day for probiotics [39]. Therefore, considering the bacteria loading achieved in the enteric coatings and the bacteria counting obtained, these formulations result a good alternative to improve the probiotic effect and points to Eudragit and Eudraguard as potential materials for the successful delivery of probiotics to the target tissue.

### 3.2. In Vitro Biological Studies

Microbiological assays were performed in order to evaluate bacteria viability under the synthesis conditions. The evaluation of the resistance of free live bacteria (*E. coli* as Gram negative [25] and *L. acidophilus* as Gram positive bacteria models) under gastric and intestinal conditions showed that *E. coli* bacteria did not survive under simulated gastric conditions, whereas for *L. acidophilus* a 4 log reduction was observed compared to the control counts (from 6.7·10^8^ to 9.3·10^4^ CFU/ml), displaying in both cases significant differences (*p* < 0.05) between encapsulated (Figure 2) and not encapsulated groups (Figure S4). Bacterial cell counts in the same order of magnitude were obtained when both bacteria were immersed under simulated intestinal conditions. As it was demonstrated, acid resistant bacteria (i.e., *L. acidophilus*) remained viable under gastric conditions but having a 4 log reduction, which is under the minimum recommended dose indicated above (10^6^ CFU/mL) [38]. However, it is important to point out that the encapsulation of bacteria has several advantages compared to the administration of live bacteria (e.g., in dairy products such as yogurt) including an easier storage, shelf life, and handling and a more controlled targeted release at specific locations of the small intestine.

**Figure 5:**
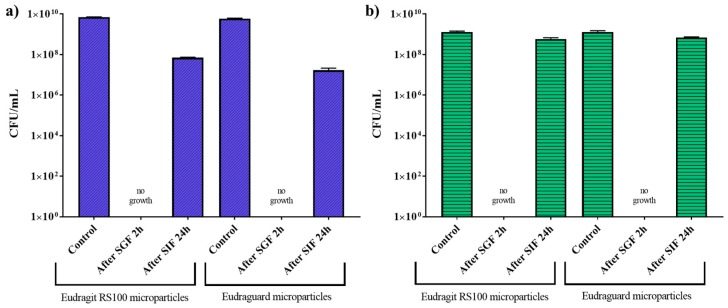
Bacterial count rate after treatment with simulated gastric (SGF) and intestinal (SIF) fluids of coated *Escherichia coli* (**a**) and *Lactobacillus acidophilus* (**b**).

Figure 5. shows the bacterial count rate after immersing the enteric-coated microparticles in simulated gastric fluid for 2 h and then in simulated intestinal fluid (in this case TSB at a pH of 7.4) for 24 h. Bacteria were not detected in the supernatants of the media recovered after gastric incubation. However, after intestinal incubation bacterial cell counts of 10^7^ CFU/mL for *E. coli* and 10^8^ CFU/mL for *L. acidophilus* were measured.

Separate experiments were performed to evaluate the bacteria growth kinetics over time by immersing the microparticles in just simulated intestinal fluid to find out how long it takes for the encapsulated bacteria to grow after a simulated digestion. The results showed a 6 h lag phase in which the bacteria adapted themselves to the growth conditions and then exponential phase counts (i.e., 10^7^ CFU/mL) were obtained after 24 h of incubation which represented a 2 log reduction compared to the counts retrieved for the control samples (i.e., 10^9^ CFU/mL).

**Figure 6:**
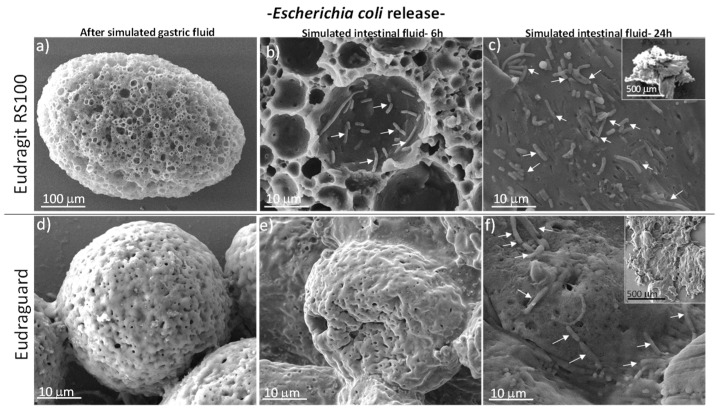
SEM micrographs of Eudragit RS 100 (**a**–**c**) and Eudraguard (**d**–**f**) microparticles loaded with *Escherichia coli* and immersed in simulated gastric (**a**,**d**) and intestinal fluids (**b**,**c**,**e**,**f**). White arrows depict the location of bacteria.

**Figure 7:**
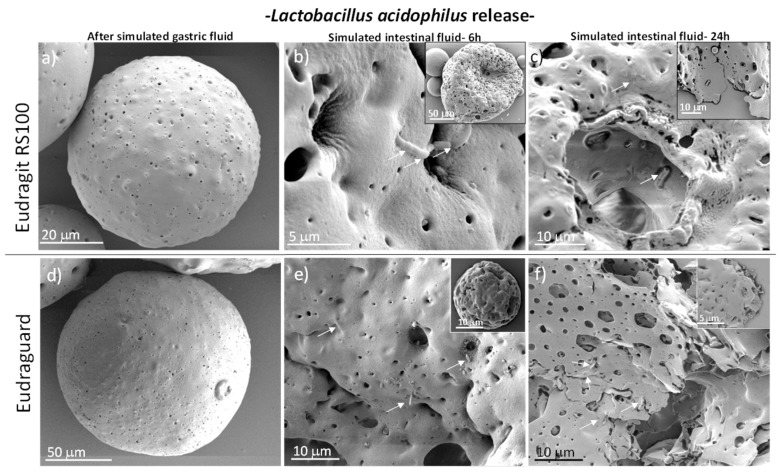
SEM micrographs of Eudragit RS 100 (**a**–**c**) and Eudraguard (**d**–**f**) microparticles loaded with *Lactobacillus acidophilus* and immersed in simulated gastric (**a**,**d**) and intestinal fluids (**b**,**c**,**e**,**f**). White arrows depict the location of bacteria.

The chemical resistance of the enteric microparticles under gastric conditions is depicted in Figure 6 and Figure 7. In the same figures, the partial erosion of the polymeric matrix under simulated intestinal conditions is depicted. As expected, the microparticles remained insoluble but they eroded becoming permeable releasing the encapsulated bacteria. The process that controls the erosion of both Eudragit and Eudraguard is similar and corresponds to a surface erosion in which the polymer degrades from the exterior surface and the inside of the material does not degrade until all the surrounding material around it has been degraded. Hence, despite the appearance of the external surface for both polymers, this surface is rapidly attacked under simulated intestinal conditions and erodes leaving a porous structure behind (Figure 6 and Figure 7) being the pores much larger than the bacteria size. Hence, no physical restrictions for the diffusion of the bacteria outwards are envisaged.

Additionally, we stored the resulting bacteria loaded microparticles collected after lyophilization under room conditions and then they were reconstituted 4 days, 1 month, and 5 months later. Bacterial cell countings (Figure S5) revealed that the bacteria remained alive and count levels were in the same order of magnitude than the ones achieved after immediately reconstituted after synthesis. In addition, these bacteria countings were found statistically significant (*p* ≤ 0.05) compared to the non-encapsulated bacteria, highlighting the effective protection of Eudragit RS 100 and Eudraguard coatings for the bacteria tested and their suitability for the targeted delivery of probiotics to the target organ.

**Figure 8:**
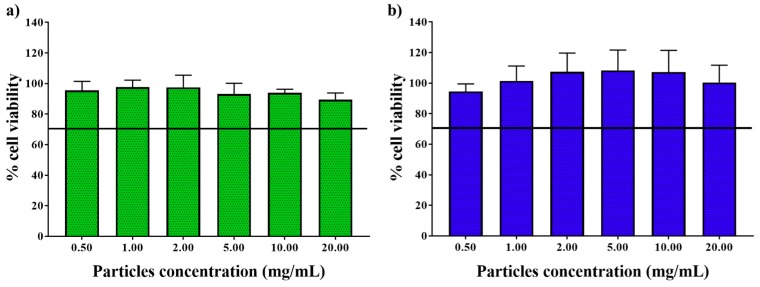
Cell viability in Caco-2 cells of Eudragit RS 100 (**a**) and Eudraguard (**b**) microparticles after incubation for 24 h. Data are presented as mean ± SD (N=4). The black line sets the biocompatibility threshold percentage (70% viability).

In order to study the cytotoxic effect, an in vitro viability assay was performed on Caco-2 cells because their phenotype mimics the enterocytes lining the small intestine. As the results shown (Figure 8), viability percentages observed were over 90% of viability at the whole range of tested concentrations (0.5–20 mg/mL), comparing them to the ones obtained with untreated cells. The international standard ISO 10993–5 dictates that a material is not cytotoxic when cell viability does not decrease more than 70% respect to the untreated cell viability. According to that, both Eudraguard and Eudragit RS 100 microparticles were cytocompatible at doses up to 20 mg/mL, being suitable for oral therapeutic administration.

As far as we know, methacrylic acid and methyl methacrylate-based polymers cellular effects have been studied on different cellular lines, on human hepatoma cell line (HepG2) [40], adenocarcinoma cell line of the human colon cells (SW480) [41], fibroblast cells (NIH/3T3) [42], with no adverse cytotoxicity effects shown. In fact, Eudragit is included in the FDA Inactive Ingredients Guide [43]. On the other hand, only a study has been developed regarding the biological implications of Eudraguard biotic [2] as material (not micro- nor nanoparticulated), not showing genotoxic effects on different mammalian cells nor inducing reverse mutation on *E. coli*. However, in all the reports mentioned, the doses tested were much lower than the ones here studied.

## 4. Conclusions

HIPE can be used to produce Eudragit RS 100 and Eudraguard biotic microparticles containing Gram negative and Gram positive bacteria models (*E. coli* and *L. acidophilus*, respectively). The synthesis protocol, polymers, and reagents used do not impair bacterial viability and the loading efficiency reached was as high as 2.8 wt %. The particle sizes obtained (212.0–293.6 µm for Eudragit RS 100 and 71.2–95.0 µm for Eudraguard loaded with *E. coli* and and *L. acidophilus*) are in the required range to achieve large residence time in the intestine while avoiding systemic absorption. Under simulated gastric conditions the microparticles remained intact but under intestinal simulated conditions the microparticles released their cargo. Viable bacteria (only a 2 log reduction compared to the control for *E. coli* and 1 log reduction for *L. acidophilus*) were recovered from the microparticles after incubating the supernatants retrieved from the immersion of the enteric microparticles in gastric and intestinal fluids, exerting bacteria countings in the range or higher than those recommended for probiotics [38,39]. Both pH-independent polymers formulated as microparticles remained insoluble under intestinal conditions but their surface eroded and permeabilized releasing the contained microbial cargo. Enteric microparticles showed excellent cytocompatibility on Caco-2 cells in concentrations up to 20 mg/mL, confirming their suitability for oral therapeutic administration. Our results highlight the potential use of these polymers as enteric coatings for the targeted delivery of probiotics, enhancing the efficiency of bacteria release in the target tissue, especially in the case of Eudraguard biotic, which, as far as we know, this is the first time that has been shown as suitable coating for drug delivery.

## Supplementary Materials

The following are available online at https://www.mdpi.com/2073-4360/11/10/1668/s1, Table S1: Parameters modified in order to optimize the synthesis conditions to obtain highly homogeneous and monodisperse particle size distributions. Figure S1: Fluorescein release after immersion in simulated gastric fluid and simulated intestinal fluid. Figure S2: Morphology of *Escherichia coli* S17 and *Lactobacillus acidophilus*. Figure S3: Thermogravimetric analysis data showing weight loss of the enteric polymers and bacteria (*Lactobacillus acidophilus*). Figure S4: Free bacteria viability test results showing bacterial cell counts of *Escherichia coli* and *Lactobacillus acidophilus* after immersing them in simulated gastric and/or intestinal fluids for 2 h and 24 h, respectively. Figure S5: Bacteria viability test results of the long-term stability study of lyophilized Eudraguard and Eudragit-coated bacteria after different times showing bacterial cell counts of *Escherichia coli* and *Lactobacillus acidophilus* after immersing them in simulated gastric and intestinal fluids.
