# Supplementary material for: Targeted Release of Probiotics from Enteric Microparticulated Formulations
Source: Polymers (Basel). 2019 Oct 13;11(10):1668. doi: 10.3390/polym11101668 (PMC6835770; doi:10.3390/polym11101668)
Supplement: Supplementary file 1 [file polymers-11-01668-s001.pdf]

polymers

Supplementary Materials: Targeted release of probiotics from enteric microparticulated formulations

Cristina Yus, Ruben Gracia, Ane Larrea, Vanesa Andreu, Silvia Irusta, Victor Sebastian, Gracia Mendoza, Manuel Arruebo

Table S1. Parameters modified in order to optimize the synthesis conditions to obtain highly homogeneous and monodisperse particle size distributions.

Solvent optimization.

Polymer	mg	Solvent	Volume	Surfactant	Internal phase
EuRS100	100	Ethyl acetate	2.5 mL	PVA 1% (w/v)	1 mL H_2O milliQ
EuRS100	100	Dichloromethane	2.5 mL	PVA 1% (w/v)	1 mL H_2O milliQ
EuRS100	100	Chloroform	2.5 mL	PVA 1% (w/v)	1 mL H_2O milliQ
Eudraguard	100	Ethyl acetate	2.5 mL	PVA 1% (w/v)	1 mL H_2O milliQ
Eudraguard	100	Dichloromethane	2.5 mL	PVA 1% (w/v)	1 mL H_2O milliQ
Eudraguard	100	Chloroform	2.5 mL	PVA 1% (w/v)	1 mL H_2O milliQ

Surfactant optimization.

Polymer	mg	Solvent	Volume	Surfactant	Internal phase
EuRS100	100	Dichloromethane	2.5 mL	PVA (HMW) 1% (w/v)	1 mL H_2O milliQ
EuRS100	100	Dichloromethane	2.5 mL	PVA (LMW) 1% (w/v)	1 mL H_2O milliQ
EuRS100	100	Dichloromethane	2.5 mL	Pluronic 1% (w/v)	1 mL H_2O milliQ
EuRS100	100	Chloroform	2.5 mL	PVA (HMW) 1% (w/v)	1 mL H_2O milliQ
EuRS100	100	Chloroform	2.5 mL	PVA (LMW) 1% (w/v)	1 mL H_2O milliQ
EuRS100	100	Chloroform	2.5 mL	Pluronic 1% (w/v)	1 mL H_2O milliQ
Eudraguard	100	Dichloromethane	2.5 mL	PVA (HMW) 1% (w/v)	1 mL H_2O milliQ
Eudraguard	100	Chloroform	2.5 mL	PVA (HMW) 1% (w/v)	1 mL H_2O milliQ

Polymer concentration optimization.

Polymer	mg	Solvent	Volume	Surfactant	Internal phase
EuRS100	25	Dichloromethane	2.5 mL	PVA (HMW) 1% (w/v)	1 mL H_2O milliQ
EuRS100	50	Dichloromethane	2.5 mL	PVA (HMW) 1% (w/v)	1 mL H_2O milliQ
EuRS100	75	Dichloromethane	2.5 mL	PVA (HMW) 1% (w/v)	1 mL H_2O milliQ
EuRS100	100	Dichloromethane	2.5 mL	PVA (HMW) 1% (w/v)	1 mL H_2O milliQ
EuRS100	150	Dichloromethane	2.5 mL	PVA (HMW) 1% (w/v)	1 mL H_2O milliQ
EuRS100	100	Chloroform	2.5 mL	PVA (HMW) 1% (w/v)	1 mL H_2O milliQ
Eudraguard	100	Dichloromethane	2.5 mL	PVA (HMW) 1% (w/v)	1 mL H_2O milliQ
Eudraguard	100	Chloroform	2.5 mL	PVA (HMW) 1% (w/v)	1 mL H_2O milliQ
Eudraguard	100	Chloroform	2.5 mL	PVA (HMW) 1% (w/v)	1 mL H_2O milliQ

Evaporation method optimization.

Polymer	mg	Solvent	Volume	Evaporation
EuRS100	100	Dichloromethane	2.5 mL	Rotary evaporator 30 min, 35 °C
EuRS100	100	Dichloromethane	2.5 mL	Room temperature
EuRS100	100	Dichloromethane	2.5 mL	Magnetic stirring 600 rpm, 3 h
EuRS100	100	Chloroform	2.5 mL	Magnetic stirring 600 rpm, 3 h
Eudraguard	100	Dichloromethane	2.5 mL	Magnetic stirring 600 rpm, 3 h
Eudraguard	100	Chloroform	2.5 mL	Magnetic stirring 600 rpm, 3 h

* All the synthesis includes PVA (HMW) 1% (*w*/*v*) and 1 mL H_2O milliQ as internal phase.

Synthesis method optimization.

Polymer	mg	Solvent	Volume	Evaporation
EuRS100	100	Dichloromethane	2.5 mL	Sonication 30 seg, 40% amplitude
EuRS100	100	Dichloromethane	2.5 mL	Magnetic stirring 5 min, 800 rpm
EuRS100	100	Chloroform	2.5 mL	Sonication 30 seg, 40% amplitude
EuRS100	100	Chloroform	2.5 mL	Magnetic stirring 5 min, 800 rpm
Eudraguard	100	Dichloromethane	2.5 mL	Sonication 30 seg, 40% amplitude
Eudraguard	100	Chloroform	2.5 mL	Sonication 30 seg, 40% amplitude
Eudraguard	100	Dichloromethane	2.5 mL	Magnetic stirring 600 rpm, 3 h
Eudraguard	100	Chloroform	2.5 mL	Magnetic stirring 5 min, 800 rpm

* All the synthesis includes PVA (HMW) 1% (*w*/*v*) and 1 mL H_2O milliQ as internal phase.

Bacteria survival optimization.

Polymer	mg	Solvent	Volume	Evaporation
EuRS100	100	Dichloromethane	2.5 mL	Ice bath
EuRS100	100	Dichloromethane	1 mL	Internal phase increased (4 mL)
EuRS100	100	Dichloromethane	1 mL	Ice bath + internal phase increased (4 mL)
EuRS100	100	Chloroform	2.5 mL	Ice bath
EuRS100	100	Chloroform	1 mL	Internal phase increased (4 mL)
EuRS100	100	Chloroform	1 mL	Ice bath + internal phase increased (4 mL)
Eudraguard	100	Chloroform	2.5 mL	Ice bath + internal phase increased (4 mL)

* All the synthesis includes PVA (HMW) 1% (*w*/*v*) and bacteria in culture medium as internal phase.

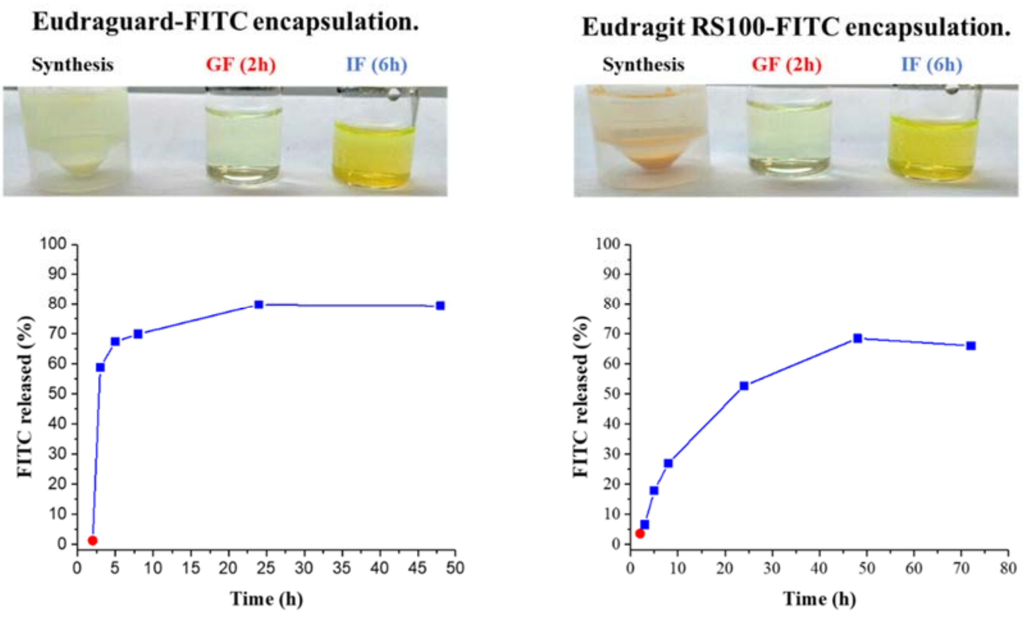

Figure S1. Fluorescein release after immersion in simulated gastric fluid (2 h, red circle) and simulated intestinal fluid (48 h-72 h, blue square). Data obtained by absorbance measurements.

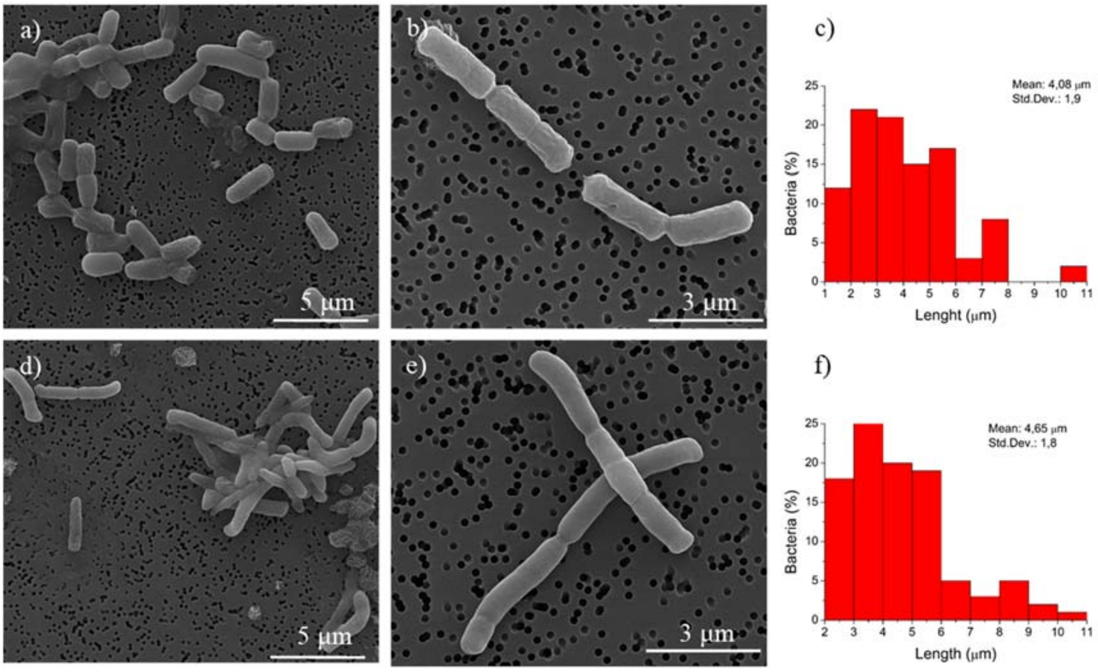

Figure S2. Morphology of *Escherichia coli S17* (a, b) and *Lactobacillus acidophilus* (ATCC® 4356™) (d, e). Histograms representing the bacterial length profiles, *E. coli* (c), *L. acidophilus* (f).

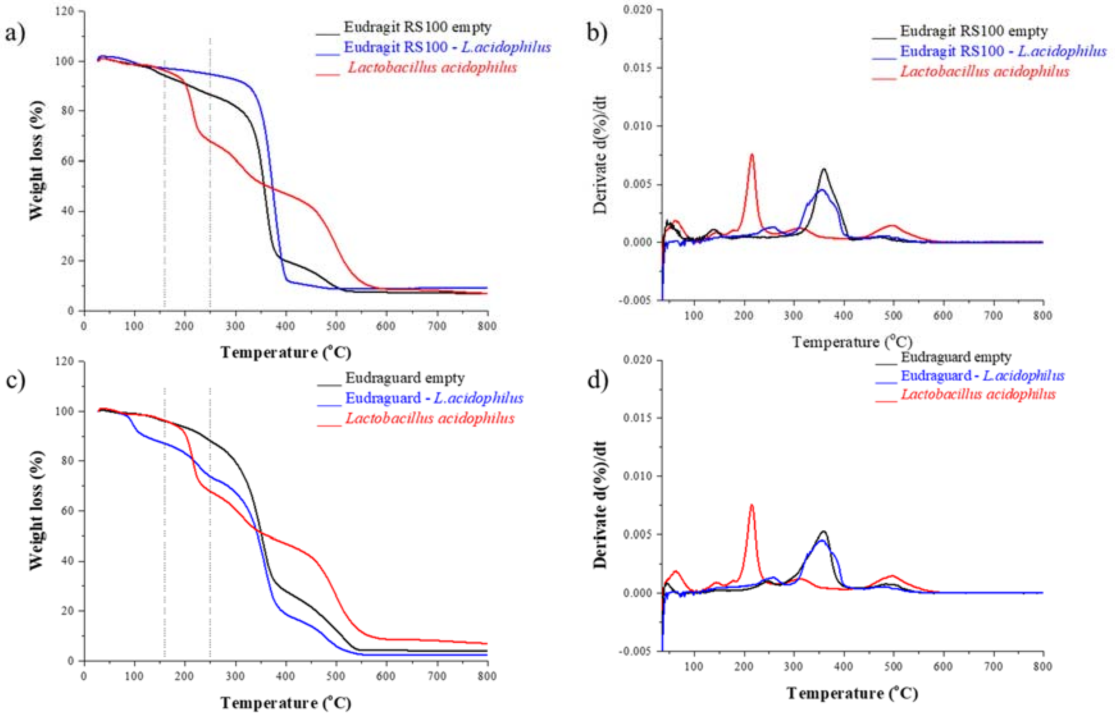

Figure S3. Thermogravimetric analysis data showing weight loss of the enteric polymers and bacteria (*Lactobacillus acidophilus*). The first derivative is also plotted in each case. a) Thermogravimetric analysis data of bacteria encapsulated into Eudragit RS 100 microparticles. b) First derivate of Eudragit RS 100 thermogravimetric analysis. c) Thermogravimetric analysis data of bacteria encapsulated into Eudraguard microparticles. d) First derivate of Eudraguard thermogravimetric analysis.

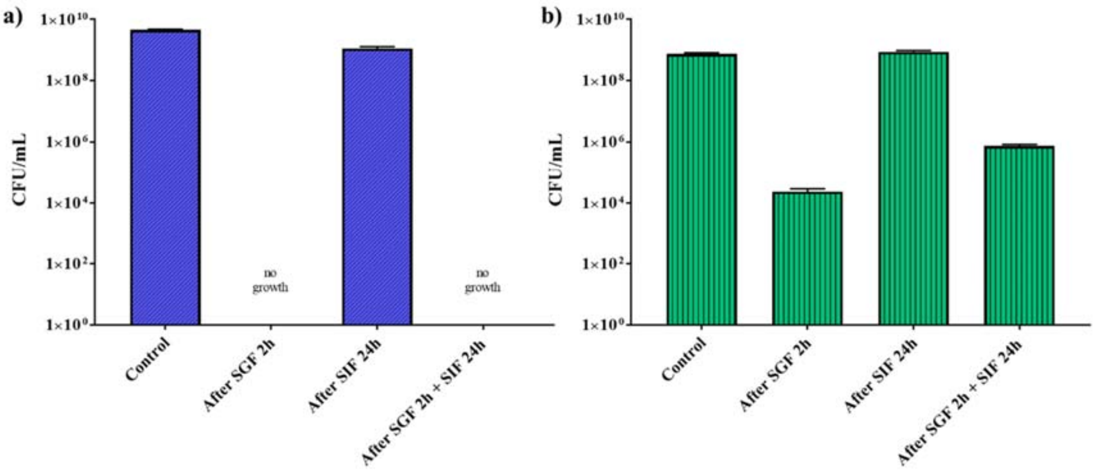

Figure S4. Free bacteria viability test results showing bacterial cell counts of *Escherichia coli* (a) and *Lactobacillus acidophilus* (b) after immersing them in simulated gastric (SGF) and/or intestinal (SIF) fluids for 2h and 24h, respectively. Results were obtained from three independent syntheses and bacteria encapsulation assayed in triplicate.

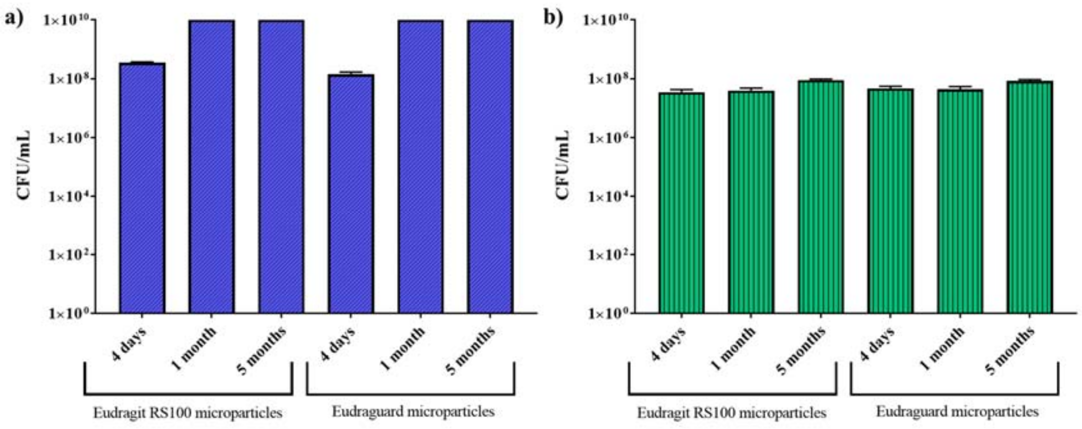

Figure S5. Bacteria viability test results of the long-term stability study of lyophilized Eudraguard and Eudragit-coated bacteria after different times showing bacterial cell counts of *Escherichia coli* (a) and *Lactobacillus acidophilus* (b) after immersing them in simulated gastric and intestinal fluids. Results were obtained from three independent syntheses and bacteria encapsulation assayed in triplicate.
